# Causes of moral distress among midwives: A scoping review

**DOI:** 10.1177/09697330241281498

**Published:** 2024-09-27

**Authors:** Michael Rost, Caterina Montagnoli, Johanna Eichinger

**Affiliations:** 27209 University of Basel; 27209 University of Basel; University of Applied Sciences and Arts of Western Switzerland; 27209 University of Basel

**Keywords:** Moral distress, midwifery, ethics, birth, pregnancy

## Abstract

Numerous studies have evidenced moral distress among midwives; however, to date no research synthesis on causes of moral distress among midwives has been conducted. A scoping review was carried out to identify, comprehensively map, and categorize possible causes of moral distress among midwives, and to identify knowledge gaps. Six data bases were searched using Boolean logic. To be included, studies had to (a) present empirical findings on (b) causes of moral distress (c) among midwives (d) in English, German, French, or Italian. We included a final set of 43 studies. The vast majority of studies came from high-income countries (83.7%) and used a qualitative approach (69.8%); 48.8% of the studies were published in the past 5 years. Identified single reasons of moral distress were grouped into eight broader clusters, forming a coherent framework of reasons of moral distress: societal disregard, contemporary birth culture, resources, institutional characteristics, interprofessional relationships, interpersonal mistreatment of service users, defensive practice, and challenging care situations. These clusters mostly capture moral distress resulting from a conflict between external constraints and personal moral standards, with a smaller proportion also from an intraindividual conflict between multiple personal moral standards. Despite projected increases in demand for midwives, the midwifery workforce globally faces a crisis and is experiencing substantial strain. Moral distress further exacerbates the shortage of midwives, which negatively affects birth experiences and birth outcomes, ultimately rendering it a public health issue. Our findings offer points of leverage to better monitor and alleviate moral distress among midwives, contributing to reducing attrition rates and improving birth experiences and birth outcomes. Further research is essential to explore the issue of ecological moral distress, develop evidence-based interventions aimed at alleviating moral distress among midwives, and evaluate the effects of both individual and system-level interventions on midwives, intrapartum care, and service users’ outcomes.

## Introduction

In 1984, Jameton defined the concept of moral distress as the psychological distress of being in a situation in which a person is constrained from acting on what the person knows to be right.^1^ Such situations of being prevented from taking the known morally correct action are often referred to as moral constraint and lie at the core of the so-called narrow definition of moral distress.^2,3^ In 2017, in light of the growing literature on moral distress,^4–6^ Jameton acknowledged that significant questions concerning the definition of moral distress have arisen.^7^ For example, is moral distress not just psychological distress^8,9^? Is the impossibility to act a necessary criterion for moral distress^10,11^? Do practitioners need to know the morally right thing to do (i.e., moral certainty), or is it simply about their opinion^10^? Responding to the increasingly scrutinized conceptual vagueness surrounding moral distress, various authors have called for a refinement of the concept of moral distress, for example, by not considering moral constraint a necessary condition of moral distress, or by broadening the concept and considering moral conflicts and dilemmata (i.e., intra-individual clash of moral values regarding the right action) a potential cause of moral distress.^8,12^ In an attempt to reconcile the narrow definition and refined definitions by other authors, Monteverde proposed to distinguish two psychological reactions to an ethically challenging situation: first, moral distress for situations of compromised moral agency or moral constraint (e.g., being prevented from carrying out the right action), and, second, moral discomfort for situations in which moral agency is burdensome but not (fully) compromised or constrained (e.g., conflicting own values).^3^ At a normative level, moral distress corresponds to situations of moral complicity (i.e., being involved in or an accomplice of a morally wrong action) characterized by a conflict *with* a moral value and moral discomfort corresponds to situations of moral complexity characterized by a conflict *between* moral values.^3^

Examining the relationships between various definitions of moral distress in the nursing literature, Deschenes and colleagues’ concept clarification of moral distress distinguishes the following elements of moral distress: (1) antecedents are events which occurred prior to the morally distressing situation (e.g., lack of resources and nurses’ belief framework), (2) internal (e.g., nurses’ moral sensitivity and feelings of powerlessness) and external attributes of moral distress (e.g., power structures and hospital policies) are characteristics which contributed to the morally distressing situation at the time of the event, and (3) consequences of moral distress exist at multiple levels of the healthcare system (e.g., emotional and physical consequences, reduced job satisfaction, and leaving a profession).^13^ A midwifery-specific concept clarification also identified antecedents and consequences of moral distress, and delineated three core attributes of moral distress, which reflect Jameton’s definition: moral (in)actions, conflicting needs, and negative emotions.^14^

Numerous factors render midwifery care of increased bioethical complexity, such as the necessity to take into account both the birthing person’s and fetal interests, structural factors undermining the birthing person’s autonomy, socio-cultural values permeating the birth context, or difficulties related to the application of informed consent and to the assessment of decisional capacity.^15,16^ Maneuvering this bioethical complexity is further aggravated by midwives’ challenging working conditions (e.g., lack of influence and recognition, negative and unsupportive facility cultures, and organizational and occupational sources of stress).^17–21^ It is hence unsurprising that midwives frequently experience moral distress. In light of this, the World Health Organization and other international organizations call for better working conditions of midwives.^22,23^

Morally relevant events that evoke moral distress involve the inability to provide adequate midwifery care due to external constraints (e.g., lack of funding and pandemic restrictions),^24–26^ conflicting values and philosophies between midwives and medical professionals as well as associated epistemic disavowal of the former,^20,27,28^ power asymmetries and professional hierarchies,^25,27,29^ oppressive practice laws,^28^ or routine midwifery tasks such as termination of pregnancy, and newborn screening.^20,30–32^ As a result, high rates of midwives choose to leave the profession, causing high turn-over rates, a loss of institutional memory, and shortages of midwives.^33–35^ Ultimately, service users’ birth experiences are being and will be negatively affected, which in turn impacts on, for example, postpartum mental health,^36^ parent–child-bonding and child well-being,^37^ parental couple relationships,^38^ or the likelihood of giving birth again^39,40^ and of accessing a birth facility for birth.^41,42^ Taken together, moral distress is not only burdensome for midwives but has adverse implications at a public health level.

Although limited, the available research on midwives’ moral distress is of primal importance, since it highlights the specifically moral labor of midwives and it can help mitigating its negative effects.^4,43^ While the conceptual fuzziness of moral distress requires to proceed with caution,^4,7–11,44^ it is paramount to study the full range of possible causes of moral distress.^7,13,14,45^ However, no research synthesis on causes of moral distress among midwives has been carried out yet. As such, the aims of this study were to identify, comprehensively map, and categorize possible causes of moral distress among midwives that have been described in empirical studies, and to identify knowledge gaps. Our findings provide points of leverage to better monitor and alleviate moral distress among midwives, contributing to lower rates of midwives leaving the job and ultimately to better care experiences by service users.

## Methods

Scoping reviews are an approach to evidence synthesis used to determine the scope and volume of a body of literature on a given topic and to provide an overview of its focus. Particularly, they are a tool to map and identify an emerging body of evidence on a topic for which more specific questions have yet to be defined.^46^ Unlike systematic reviews’ emphasis on precise and clinically relevant research questions, scoping reviews apply less restrictive inclusion criteria and draw upon data from all studies regardless of design or quality.^46^ As such, the method is well suited to fully map the terrain of causes of moral distress among midwives.

### Search strategy and information sources

Besides the authors’ knowledge about moral distress, search terms were identified by, first, preliminarily searching data bases and analyzing key words and words contained in titles and abstracts, second, incorporating expert suggestions, and, third, searching on the internet. We broke down the search into “midwives” (i.e., population of interest) and “moral distress” (i.e., concept of interest). To frame the review, the Preferred Reporting Items for Systematic Reviews and Meta-Analyses were followed.^47,48^ In addition, we applied Arksey and O'Malley’s framework for scoping reviews which is composed of the following stages: identification of research question, identification of relevant studies, selection of studies, charting of data, and collating, summarizing, and reporting the results.^49^ We searched six databases, employing Boolean logic (Table 1).

**Table 1.** Search terms and search results.

No	Search terms	Matches					
		Scopus	Pub Med	PsycInfo	Web of Science	cinahl	Soc index
**1**	(“midwife*” OR “midwives”)	50.998	93.643	4.114	55.097	68.122	2.882
**2**	(“Moral distress” OR “moral dilemma*” OR “moral challeng*” OR “moral injur*” OR “moral residue” OR “moral uncertaint*” OR “moral conflict*” OR “moral constraint*” OR “moral trauma*” OR “moral stress*” OR “moral issue*” OR “moral rectitude” OR “moral responsibility*” OR “moral identity” OR “moral character*” OR “moral agency” OR “moral harassment*” OR “moral sensitivity” OR “moral heroism” OR “moral decisiveness” OR “moral stand*” OR “moral value*” OR “moral capability” OR “moral resilience” OR “moral courage” OR “moral anxiety” OR “moral suffering” OR “moral omission” OR “moral disturbance”)	26.231	9.771	10.802	27.856	5.480	12.479
**1 and 2**	**Total: 483**	**72**	**228**	**16**	**78**	**70**	**19**

Date of last search: 19.01.2023

### Eligibility criteria

To be included, studies had to (a) present empirical findings on (b) causes of moral distress (c) among midwives and (d) be written in English, German, French, or Italian. No inclusion criteria were applied to study methodology, study quality, geographical region, or publication date. To accumulate a comprehensive census of relevant literature, we intentionally defined moral distress broadly for the purpose of this scoping review, namely, as follows:

- a negative emotion (e.g., distress, stress, unease, disquiet, and cognitive-emotional dissonance);
- owing to a morally relevant event (e.g., actions, inactions, and circumstances) regarding which a midwife experiences;
- either external constraints (e.g., hospital guidelines, laws, lack of resources, and instructions of superiors) conflicting with their moral standards, ultimately hampering midwives’ ability to act morally;
- or multiple own moral standards conflicting with one another internally (e.g., respect for service users’ autonomy vs respect for fetal life), ultimately hampering midwives’ ability to act morally.^3,27,44,50–52^

Therefore, the definition of moral distress used in this review also encompasses what Monteverde has described as moral discomfort.^3^ In fact, while Monteverde stresses that in situations of moral discomfort due to multiple conflicting moral values moral agency is only burdensome—yet not compromised by external factors as in the case of moral distress^3^—here we consider both burdensome and compromised moral agency forms of limited moral agency. We decided to include both aspects as we aimed to cover a comprehensive spectrum of morally undesirable situations experienced by midwives. Similarly, we deliberately included studies that omitted to use the term “moral distress” but described experiences of midwives that matched our definition of moral distress. Lastly, following the same rationale (i.e., aiming to cover a comprehensive spectrum of morally undesirable situations), we purposely left out the contested aspects of harm (i.e., providers believing they have contributed to harm on the part of others) and of moral certainty (i.e., knowledge about the right action) as necessary criteria of moral distress. While some authors have proposed these aspects as necessary criteria of moral distress,^1,52,53^ we deliberately did not. This decision allowed us to include both studies describing moral distress due to the belief that one has contributed to the harm of others but also studies describing moral distress that does not result from this belief. Similarly, this way we could incorporate both studies describing moral distress in situations of moral certainty as well as those describing moral distress in situations without moral certainty.

### Selection process and search results

**Figure 1.**
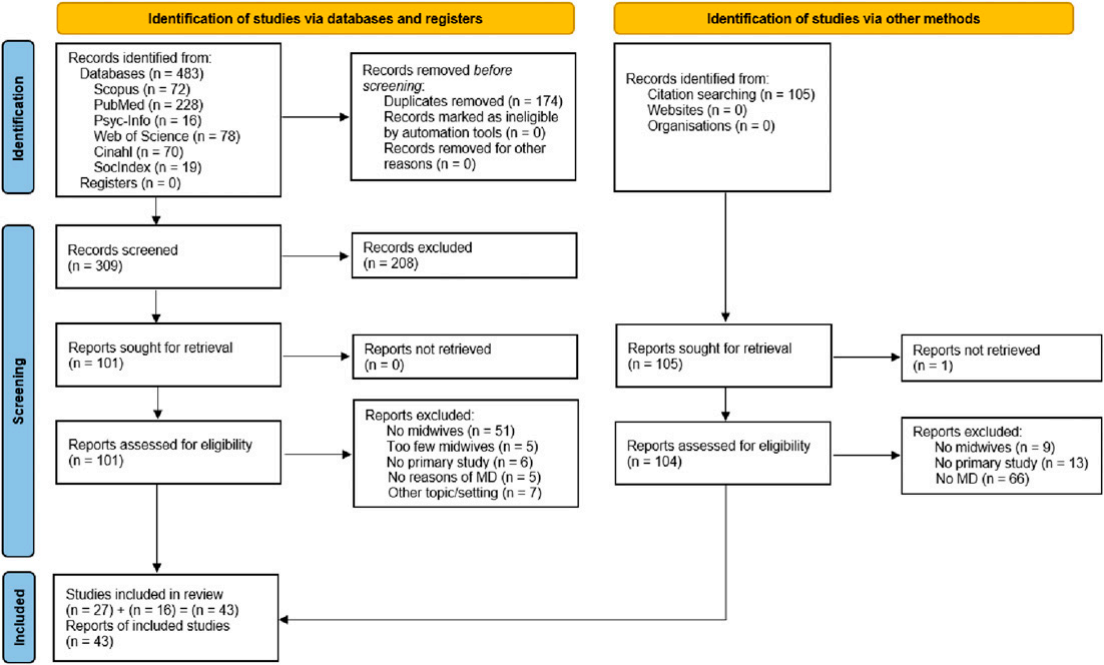
PRISMA flowchart for inclusion of studies.

The search resulted in 483 records. After de-duplication, 309 records remained, which were screened based on title and abstract. This step resulted in 101 reports, from which 20 were randomly selected and assessed for inclusion based on full-texts by two coders independently (CM and MR). Cohen’s kappa was calculated to assess interrater reliability for eligibility of inclusion. A kappa of 1.00 indicated a perfect agreement,^54^ and thus one rater (MR) assessed eligibility for inclusion of the remainder. This step resulted in 27 studies. The references of these studies were checked to identify additional records, resulting in 16 studies. Hence, the final set of included studies was composed of 43 studies (Figure 1).

### Data analysis

We developed a 27-item data extraction framework containing information in the following main areas of interest: general study characteristics (e.g., title, authors, year of publication, country of origin, and aim), study design and sample (e.g., type of analysis, age, work experience, and composition of sample), information on moral distress (e.g., moral distress explicitly mentioned, underlying definition of moral distress, description of moral distress, and causes of moral distress).

To identify recurring reasons for moral distress, evidence was coded using inductive content analysis and the analysis software MAXQDA.^55^ In this step, the specific conflict causing moral distress was reconstructed and explicitly spelled out, that is, either between external constraints and own moral standards (moral complicity) or between multiple own moral standards (moral complexity). Subsequently, identified single reasons of moral distress were grouped into broader clusters of reasons of moral distress based on significant commonalities regarding the causes of moral distress. Lastly, clusters of reasons were refined and named to build a coherent framework of reasons of moral distress among midwives.

## Results

A total of 43 papers were included in the review. The vast majority came from high-income countries (*n* = 36, 83.7%; mostly Europe: *n* = 19, 44.2%, and Australia and Oceania: *n* = 9, 20.9%), five from lower-middle income countries (11.6%; mostly Ghana: *n* = 3, 7.0%; note that one study was binational with the second country being low income, namely, Burkina Faso), and two from upper-middle income countries (4.7%; Brazil).^56^ Most studies used a qualitative approach (*n* = 30, 69.8%), eight a quantitative approach (18.6%), and five a mixed-methods approach (11.6%). Almost half of the studies were published in the past 5 years (*n* = 21, 48.8%) and more than nine out of ten since 2010 (*n* = 39, 90.7%). Samples of almost two-third of studies were exclusively composed of midwives (*n* = 27, 62.8%; two studies with nurse-midwives and four studies with midwifery students). Almost one-third explicitly mentioned the term “moral distress” (*n* = 13, 30.2%) among which nine studies provided a definition of moral distress (69.2%). Further details on characteristics of included studies are presented in Table 2.

**Table 2.** Characteristics of included studies.

1^st^ author	Year	Study aim	Country	Midwives	N	Exp	Age	Setting	Design	Moral distress
Addo^24^	2020	Explore and understand moral distress from the perspective of and as experienced by Ms	Ghana	100%	8	≤5–≥15	<35–>45	Hospital	Qual	Yes
Armour^57^	2021	Gain a deeper understanding of Ms’ role in termination of pregnancy	New Zealand	100%	8	3–>40	22–60	Hospital	Qual	No
Beck^58^	2015	Determine the prevalence and severity of secondary traumatic stress and explore descriptions of experiences attending traumatic births	USA	100%	473	*M* = 16.7 *SD* = 10.0	*M* = 50.4 26–77	Hospital	Mixed	No
Beck^59^	2022	Investigate the presence of symptoms of moral injury	USA	43.1%	75	-	-	Hospital	Qual	No
Boakye^25^	2021	Explore the moral habitability of obstetric settings and how this influences Ms’ moral agency	Ghana	73.3%	22	≥0.5	24–56	Hospital	Mixed	Yes
Boakye^60^	2022	Provide an in‐depth analysis of the influence of resource scarcity on care and practice	Ghana	73.3%	22	≥0.5	24–56	Hospital	Mixed	No
Callwood^61^	2018	Explore how students describe their ‘values journey’ following exposure to clinical practice	UK	40.5%	17	0	18–42	Hospital	Qual	No
Callwood^62^	2019	Explore how students describe their “values journey” after completing 2^nd^ year following exposure to clinical practice	UK	57.1%	16	0	18–42	Hospital	Qual	No
Cignacco^63^	2002	Investigate how Ms view the termination of pregnancy because of a pathological fetal condition and to clarify their ethical position	Switzerland	100%	13	Diverse^a^	27–53	Hospital	Qual	No
Craswell^64^	2014	Examine the factors that influence Ms when entering perinatal data	Australia	-	-	-	-	Hospital	Qual	No
Fontein-Kuipers^65^	2018	Examine the experience of dilemmas involving conflicting values, triggered by practice to better engage Ms in (reflective) practice	Netherlands	100%	11	*M* = 13 1–28	*M* = 37 22–59	M-practice	Qual	No
Foster^27^	2021	Explore Australian Ms’ experience and consequences of moral distress	Australia	100%	14	0–21	-	M-practice	Qual	Yes
Fumagalli^26^	2022	Explore Ms’ experiences of providing care during the COVID-19 pandemic	Italy	100%	15	*M* = 13.13 2–31	*M* = 36.8 25–54	Hospital	Qual	Yes
Garel^66^	2002	Explore the conflicts and ethical problems experienced in prenatal diagnosis and termination of pregnancy	France	63.8%	30	-	-	Hospital	Qual	No
Garel^31^	2007	Study the clinical, emotional, and moral difficulties that Ms encounter while performing termination of pregnancy	France	100%	87	<6–>16	20–>50	Hospital	Quant	Yes
Groothuizen^67^	2019	Explore the “values journey” of student Ms across the 3 years of their education program	UK	56.0%	14	0	0	Hospital	Qual	No
Guidera^68^	2021	Explore the extent to which adverse obstetric and gynecologic outcomes resulted in litigation proceedings	USA	100%	1340	-	*M* = 50.6	Hospital	Quant	No
Hadjigeorgiou^69^	2014	Explore the perceptions of Ms as client advocates for normal birth	Cyprus	100%	20	*M* = 22.0	-	Hospital	Qual	No
Harvie^34^	2019	Determine the incidence of Ms indicating their intention to leave the profession and explore the reasons for this decision	Australia	100%	1037	*M* = 16.5	46.4	Hospital	Mixed	No
Hood^70^	2010	Describe Ms’ experiences of external review of obstetric services, involvement in legal proceedings, and its impact	Australia	100%	16	0–30	24–55	Hospital	Qual	No
Jaffre^71^	2021	Understand Ms’ experiences with caregiving	Benin B. Faso	100%	24	Diverse^a^	24–66	Health services	Qual	No
Jefford^72^	2022	Establish Ms’ perceptions of the value of workshops designed to empower their decision-making and leadership skills	Australia	100%	31	<2–>20	20–>60	Hospital	Qual	Yes
Kane^73^	2022	Explore the psychological experience of students who had been deployed to work in the NHS during the pandemic	UK	35%	19	0	*M* = 30.9 *SD* = 8.7	Hospital	Quant	Yes
Kerkman^74^	2019	Study the prevalence among Ms of experiencing work-related traumatic events and consequently developing mental disorders	Netherlands	100%	691	*M* = 10.0 *IQ* = 7–16	20–>65	Mixed	Quant	No
Lindström^75^	2007	Gain knowledge about Ms’ experiences of working with termination of pregnancy and perception of women’s motives	Sweden	100%	216	-	32–65	Mixed	Quant	No
Lindström^76^	2011	Elucidate Ms’ experiences, perceptions, and interactions working in abortion services	Sweden	37.5%	15	<1–>30	23–62	Hospital	Qual	No
Marsh^77^	2020	Explore Ms’ experiences of moral distress when providing care to women whose babies were removed at birth	UK	66.6%	8	-	-	-	Qual	Yes
Memmott^78^	2022	Explore Ms’ experiences working on the frontlines of the pandemic	Canada	100%	13	-	-	M-practice	Qual	Yes
Mizuno^32^	2011	Understand Ms’ experiences and attitudes toward their job	Japan	100%	11	5–22	24–48	Hospital	Qual	Yes
Mizuno^79^	2013	Explore the relationship between professional quality of life and emotion work and stress factors related to abortion care	Japan	33.7%	86	*M* = 18.6 *SD* = 9.6	*M* = 43.3 *SD* = 10.3	Hospital	Qual	No
Narchi^80^	2017	Describe the experiences of Ms and the characteristics of their work within the national health system	Brazil	100%	10	*M* = 1.3	*M* = 26.8	Mixed	Qual	No
Nunes^81^	2016	Explore beliefs, values, experiences, and practices related to the provision of care with the implementation of a new facility structure	Brazil	45.5%	10	10–26^2^	28–46^b^	M-unit in hospital	Qual	No
Oelhafen^20^	2019	Gain insights into ethical issues Ms encounter, key competences and resources they consider indispensable, and phenomena linked to moral distress	Switzerland	80%	8	-	*M* = 43.6 26–56	Mixed	Qual	Yes
Oelhafen^29^	2020	Understand the structure of ethical issues and moral competences, and assess the factors leading to moral distress and its negative consequences	Switzerland	100%	254	*M* = 13.7^c^ *SD* = 10.6^c^	*M* = 40.1^c^ *SD* = 11.1^c^	Mixed	Quant	Yes
Oerlemans^30^	2017	Study the moral problems Ms encounter, as well as of the ways in which they tend to respond to them	Netherlands	11.1%	4	-	-	Hospital	Qual	Yes
Reiger^82^	2013	Examine the tension between professional discourse and everyday working realities	Australia	64.7%	150	-	-	Hospital	Qual	Yes
Rice^83^	2013	Explore the experiences of Ms witnessing traumatic birth	Australia	100%	10	*M* = 5.5 1-23	-	Mixed	Qual	No
Robertson^84^	2016	Explore how Ms׳ personal involvement in clinical negligence litigation affects their practice	UK	100%	22	-	-	-	Qual	No
Surtees^85^	2010	Analyze the actions between women and Ms that constitute midwifery partnerships	New Zealand	40.5%	40	-	-	-	Qual	No
Thumm^28^	2022	Understand Ms’ experiences practicing within the healthcare system	USA	100%	1081	*M* = 14.0 *SD* = 10.1	*M* = 50.3 *SD* = 19.5	Mixed	Qual	Yes
Toohill^86^	2019	Determine prevalence of birth-related trauma and fear in Ms and to describe Ms’ experiences of birth- related trauma and/or fear	Australia	100%	249	*M* = 18.3 *SD* = 11.0	*M* = 49.0 *SD* = 9.5	Mixed	Mixed	No
Zareba^87^	2020	Assess emotional complications in Ms participating in pregnancy termination	Poland	100%	181	*M* = 16.73 *SD* = 8.63	*M* = 40.8 *SD* = 8.6	Hospital	Quant	No
Zolala^88^	2018	Determine the severity and frequency of moral distress in Ms working in birth centers	Iran	100%	180	*M* = 6.43 *SD* = 0.89	*M* = 31.4 *SD* = 8.1	Birth centers	Quant	Yes

Note. Moral distress = moral distress; M = midwife; Qual = qualitative Quant = quantitative; Mixed = mixed methods; B. Faso = Burkina Faso; Exp. = years of experience; *M* = mean; *SD* = standard deviation; IQ = interquartile range.

^a^Only reported as text that sample composed of different levels.

^b^Only for key-informants.

^c^For midwives (sample composed of midwives and midwifery students).

**Figure 2.**
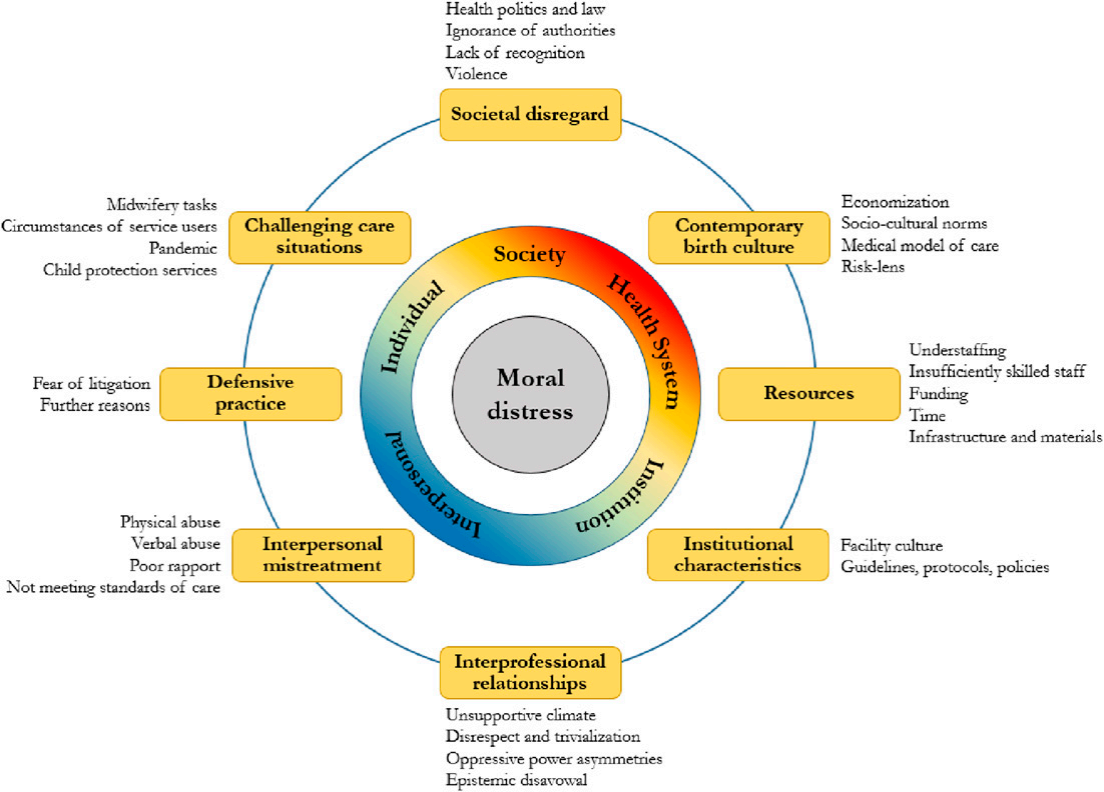
Clusters of causes of moral distress among midwives.

We identified the following eight clusters of reasons of moral distress among midwives, which will be presented in the below sections: (1) societal disregard, (2) contemporary birth culture, (3) resources, (4) institutional characteristics, (5) interprofessional relationships, (6) interpersonal mistreatment of service users, (7) defensive practice, and (8) challenging care situations (Figure 2). The identified clusters can be located at the level of the individual, of interpersonal relationships, of the institution, of the health system, and of society. Naturally, levels overlap and permeate one another (e.g., society and health system), and clusters similarly can be located at multiple levels (e.g., interprofessional relationships can be co-determined by institutional characteristics, societal aspects, and individual experiences of a midwife). While clusters (1) to (5) exclusively capture conflicts between external constraints and own moral standards (moral complicity), clusters (6) to (8) also capture conflicts between multiple own moral standards (moral complexity), mostly in the case of termination of pregnancy. A comprehensive list of specific conflicts for each cluster is provided in Table 3 in the appendix.

### Societal disregard

Societal disregard captures a plethora of instances in which midwives experience a lack of appreciation in various areas of society, such as politics, health authorities and institutions, or among service users. Societal disregard is a direct cause of moral distress among midwives. Midwives’ moral distress results from obstructive health politics and oppressive health laws regulating midwifery practice,^28,61^ and from authorities disregarding and ignoring midwives’ undesirable care experiences (e.g., violence and sexual harassment) or failing to support midwives in face of negative online reviews.^25,28,71^ Also, a lack of recognition of (parts of) their work by service users or society as a whole, leads to moral distress among midwives, who become disappointed and dissatisfied.^25,28,34,57^ Lastly, experiences of violence and aggression evoke moral distress among midwives.^25^

### Contemporary birth culture

Challenges and tensions surrounding the economization of birth care and associated health-economic imperatives lead to moral distress among midwives.^28^ Besides, various socio-cultural norms negatively affect midwifery practice. It has to be noted that some of these socio-cultural issues described in the following are likely to be limited to the respective contexts and thus are less transferable to other contexts. For example, gender-related norms systematically disempower service users and hinder them from initiating urgent interventions (e.g., because first the husband, the father-in-law, or a “fetish priest” have to agree), ultimately leaving midwives in a powerless position and with limited moral agency.^25,80^ Moreover, midwives describe how the cultural norm of high regard for physicians enables physicians to effectively change midwives’ working environment by pushing back on independent midwifery and shaping service users’ perceptions, choices, and information as well as the media portrayal of birth and midwifery.^85^ On similar lines, numerous studies refer to the so-called medical model of care as a cause of moral distress, which—as an antipode of the midwifery model of care—conflicts with midwives’ professional autonomy, midwifery-led care, and a so-called normal birth philosophy, ultimately causing dissatisfaction, powerlessness, frustration, and stress among midwives.^27,28,34,69,70,83,85,86^ Related to this, the widespread perception of birth as a high-risk endeavor and a focus on the “abnormal” are further reasons for moral distress, as they hamper midwifery care and natural birth, eliciting negative feelings and fear.^34,70,85^

### Resources

Oftentimes, a lack of resources constraining midwives in their practice causes moral distress. Accordingly, another major cause of moral distress related to resources is understaffing, which regularly hinders midwifery care and sometimes limits reproductive rights of service users.^24,25,29,32,34,58,61,67,69,78,81,82,86,88^ Besides, inexperienced and inadequately trained staff,^32,86^ limited funding directed towards midwifery,^34^ limited necessary infrastructure,^20,24,25,60,71,81,82,86^ and a lack of time^20,27,30,34,60–62,67,69,71,81,82^ are major causes of moral distress among midwives related to resources. As a consequence of time constraints, midwives cannot fully practice the values of midwifery care, have insufficient rest, sometimes have to violate reproductive rights of service users, and cannot engage in reciprocal supervision and exchange among colleagues.^30,69^ These resource-related constraints cause conflicts, stress, dilemmata, struggles, anxiety, challenges, and helplessness among midwives and compromise care.

### Institutional characteristics

Various aspects of a facility’s culture cause moral distress among midwives. Facility cultures are apostrophized as “us versus them” and full of pressures to conform to the system,^27^ technocratic and professional-centered,^80^ unsupportive of raising concerns,^61^ resistant to change,^34^ or as cultures of ignorance and fear.^70,77^ Furthermore, the primacy of institutional demands and needs is described as a key attribute of many facilities (e.g., tick-box exercise, institution-focused environment, business plans, and operational flows),^28,34,77^ and facilities’ “‘eminence’-based” cultures are mainly determined by physicians’ methods of care provision (including questionable practices and values).^13,20,78,81^ As a result, midwives’ needs are neglected,^82^ birth is dehumanized,^28^ and care inevitably compromised,^61^ which in turn causes inner conflicts, powerlessness, and distress on the part of midwives.

Besides mostly unwritten and informal aspects of culture, a facility’s guidelines, policies, and protocols frequently cause moral distress among midwives, more precisely feelings of being constrained, contested, frustration, dissatisfaction, loyalty conflicts, or negative experiences of dilemmata or contradictions.^27,28,34,64,65,69,70,82,88,89^ These formal rules mainly affect midwives’ professional autonomy and their aspiration to prioritize service users’ over institutional or providers’ needs (e.g., increase in interventions), and the time actually spent with service users (e.g., time needed for entering data into electronic medical records).

### Interprofessional relationships

Moral distress among midwives often follows from a disorganized and unsupportive atmosphere in their working environments.^25,28,71,86^ Moreover, midwives regularly have to argue over their practice with medical professionals and are confronted with negative role models and mentors.^62,70^ In such a climate, midwives feel strained, anxious, and tired of battling, which ultimately undermines the quality of midwifery care. Relationships with other providers are further characterized by oppressive power asymmetries that mainly impact on midwives’ professional autonomy, the provision of midwifery care, and—in some instances—on their freedom of sexual harassment in their workplace.^20,25,27–29,34,59,61,69,71,81,86,89^ Terms used to describe these power structures are, for example, hierarchical, oppressive, patriarchal, hegemonic, authoritative, toxic, and asymmetric. Unsurprisingly, being exposed to such power differentials for long periods of time leads to midwives experiencing constraints, disempowerment, intimidation, silencing, and a loss of control.

At the level of direct interactions between midwives and other providers, the former experience disrespect and trivialization by the latter, which is primarily directed towards midwifery as a profession.^24,25,27,34,69–71,86^ As such, midwives embodying the profession of midwifery are frequently devalued, not respected, not appreciated, ridiculed, and negatively treated. With respect to midwives’ birth care competences, they are ignored, silenced, perceived as less valid, questioned, not trusted, and disqualified by other providers.^24,25,27,29,34,69,81,89^ These multifaceted experiences of epistemic disavowal bring about damaged identities, eroding confidence, and distress on the part of midwives.

### Interpersonal mistreatment of service users

The types of interpersonal mistreatment presented in the following build on the typology of mistreatment during birth developed by Bohren and colleagues.^90^ In the included studies, the following domains of interpersonal mistreatment during birth are reported to elicit moral distress among midwives, since they violate service users’ reproductive rights: physical abuse (e.g., violence),^71,80,86^ verbal abuse (e.g., harsh language, lies, and pressures),^58,59,71,83,86^ poor rapport between service users and providers (e.g., limited autonomy and dismissal of service users’ demands),^20,62,71,82,83,86^ and failure to meet professional standards of care (e.g., lack of privacy, limited choice, and unnecessary interventions).^27,28,59,69,70,81,83,86,88^ It has to be noted, that apart from one exception where midwives know about service users’ reproductive rights but nevertheless use violence themselves because they think it is necessary to facilitate the birthing process (e.g., during crowning) and because they are exhausted,^71^ moral distress is caused by mistreatment of service users by *other* midwives or providers. Passively witnessing others mistreating service users evokes feelings of being complicit, powerlessness and inability (i.e., to protect service users), and stress.

### Defensive practice

Owing to fear of litigation, midwives often provide care not primarily to benefit the service user but to avoid and reduce risks, resulting in over-treatment and over-examination.^65,67,68,70,84–86^ Such defensive practice conflicts with fundamental principles of midwifery care, hampers the possibility of advocating for service users, and destroys trust among providers. Besides defensive practice that follows from a generalized fear of litigation, other instances of defensive practices result from a perceived necessity to cover oneself because service users do not trust the midwife and thus the midwife distrusts the service users,^85^ or from a shaken belief in the birth process due to experiences of traumatic births (e.g., shoulder dystocia, prolapsed umbilical cords, and placental abruptions) which lead to a heightened “index of suspicion,”^21,58^ alertness. Lastly, midwives with an elevated Hospital Anxiety and Depression score practiced more defensively than midwives without an elevated score.^74^

### Challenging care situations

Some routine tasks of midwives cause moral distress by provoking multiple moral values that conflict with one another. The most frequent example is termination of pregnancy.^27,29,31,32,63,66,76,79,87,88^ In these situations, midwives experience an internal conflict between service users’ rights to abortion on the one hand and a right to life of the fetus on the other hand, which ultimately causes unease, burden, distress, and feelings of inadequacy. Notably, in one study midwives’ moral distress due to termination of pregnancy stems from a perceived danger of a slippery slope (i.e., a situation, once it has started, becomes worse and worse), namely, increasingly spreading eugenic values in society.^66^ Other examples of routine midwifery care that evoke moral distress are informing about newborn screenings and possibly detected diseases,^30^ service users’ preferences for elective interventions (e.g., cesarean section),^29,88^ service users’ unrealistic expectations,^28^ and service users’ autonomy to make decisions not recommended by the midwife.^86^

Besides care situations that can be considered part of midwifery care, midwives’ involvement in challenging care situations related to service users’ circumstances (e.g., being impoverished),^25,88^ the COVID-19 pandemic (e.g., pandemic restrictions and avoidance of transmission),^26,73,78^ and child protection services (e.g., removal of babies after birth) also elicit moral distress among midwives.^77^

## Discussion

To date, no comprehensive research synthesis has addressed the causes of moral distress specifically among midwives. This scoping review sought to fill this gap by identifying, mapping, and categorizing potential causes of moral distress among midwives, as documented in empirical studies, while also identifying knowledge gaps. Embracing a broad definition of moral distress to encompass a comprehensive spectrum of morally undesirable situations encountered by midwives, we finally included 43 articles in our study. From these, we extracted distinct conflicts causing moral distress, which were grouped into eight clusters building a cohesive framework elucidating the reasons for moral distress among midwives. These clusters mostly capture conflicts between external constraints and own moral standards, but to a smaller proportion also conflicts between multiple own moral standards. The presented findings might be transferable to some extent to other occupational groups, whose experiences are likely to be similar to midwives’ experiences, such as obstetric nurses or maternity nurses.

It should be noted that these clusters cannot be viewed in isolation from each other; instead, they reinforce one another and are mutually constitutive, resulting in midwives being trapped in a wheel of moral distress. For example, the contemporary birth culture, with its strong focus on the medical model of care, is clearly intertwined with the institutional characteristics, which in their policies and guidelines, but also in their facility culture, not only reflect but also bolster the contemporary birth culture. Closely interwoven with these to clusters are also the interprofessional dynamics, such as the marginalization of midwives’ expertise (epistemic disavowal), as well as the clusters defensive practices and societal disregard. The social ecological model as a comprehensive, multi-level framework aptly illustrates the complex and contextual nature of the factors that cause moral distress and offers a nuanced perspective on how different levels of the midwife’s environment, spanning from personal to societal, interact and shape behavior and experiences.^91–93^ It demonstrates that the identified clusters can be situated across various levels, which naturally intersect and permeate one another (e.g., society and health system), and clusters similarly may manifest at multiple levels (e.g., interprofessional relationships can be co-determined by institutional characteristics, societal factors, and individual experiences of a midwife). Hence, it is imperative to broaden the scope beyond individual-level factors and consider systemic influences, enabling a multidimensional understanding of moral distress among midwives that encompasses individual choices, interpersonal dynamics, community norms, institutional, and broader societal factors.

Growing concern surrounds the prevalence and consequences of moral distress among healthcare professionals more generally, with a notable increase of the body of empirical studies in recent years. Consequently, several evidence syntheses on moral distress among health professionals, excluding midwives, have also emerged, such as among physicians or nurses.^94–100^ These reviews similarly highlight the interdependence and mutual reinforcement of various causes of moral distress, which are influenced by various personal, contextual, professional, and socio-cultural factors. However, the midwifery profession encompasses several unique attributes that may subject midwives to sources of distress different from those experienced by other healthcare providers. One of these attributes is the moral complexity inherent in midwifery practice, stemming from the perceived need to balance the interests of both the birthing person and the fetus.^15,16^ Additionally, midwives’ roles, responsibilities, and skills, such as in the termination of pregnancies, contribute to this complexity.^31^ Moreover, socio-cultural values (e.g., patriarchal and racist) permeating the birth context as well as conflicting values and philosophies between midwives and medical professionals along with the associated epistemic disavowal of the former, further compound these challenges.^101–104^

Recent developments of the midwifery profession in several countries reflect the societal disregard which was identified to be a cause of moral distress. For example, in Germany, premiums for professional liability insurance for freelance midwives have surged since the start of the millennium, causing financial hardship for many.^105^ Remarkably, the significant responsibility shouldered by midwives has not led to a high-income, as in other professions, but rather soaring insurance costs that they must bear themselves. The very low compensation of midwifery stems from and expresses the societal disregard and weak advocacy for their interests. It is reasonable to assume that the same applies to midwifery as to another profession less esteemed by society, namely, nurses: here a study shows that moral distress among nurses is higher than among physicians.^106^ Similar developments are currently under way in France where midwives are gradually invested with more and more responsibilities, among others a 6-year educational journey, autonomous management of abortion care, or couples’ fertility follow-ups, without any financial recognition.^107^ Research among midwifery students in France showed that changings within the profession, such as increased responsibilities and experiences of financial hardship, greatly affect students: seven out of ten future midwives suffer from depression during their studies, with 15% eventually abandoning the profession.^108,109^ On an opposite note, in the Netherlands midwives’ autonomy in care could soon experience a setback due to the introduction of stricter regulatory frameworks that limit their independent practice due to changes in funding schemes for the healthcare insurance—threatening autonomy in decision-making in certain clinical scenarios.^110,111^ This could not only diminish their ability to practice independently but also contribute to a sense of decreased professional value among peers and within the healthcare system.^112^ These growing bureaucratic pressures and increasing oversight from healthcare institutions have led to further erosion of midwives’ autonomy in the Netherlands, complicating their work environment and exacerbating the challenges they face daily.^113,114^ These factors collectively contribute to the moral distress experienced by midwives, as their professional autonomy and respect within the community continue to be undermined.

The deep effect of resource constraints has also been shown in a recent review on midwives well-being and resilience.^115^ The same applies to the influence of contemporary birth culture, in which, among other things, a medicalized practice environment impedes midwives from practicing in alignment with their own professional beliefs and values as well as a hospital culture that runs counter to the midwifery philosophy.^115–117^ The clusters institutional characteristics, interprofessional relationships, and interpersonal mistreatment of services users are also closely linked to this. A very recent review, for example, showed that instances of disrespectful perinatal care witnessed by midwives from healthcare providers could have traumatic effects on them.^117^ Establishing a moral community that is multidisciplinary seems to be a lever for reducing the risk of moral distress among midwives.^118^

The challenging care situations mentioned frequently revolved around terminations of pregnancies, as an internal conflict between the values of guaranteeing service users’ reproductive autonomy and a perceived right to life of the fetuses involved. In several countries, including Italy, midwives and gynecologist have the right to conscientious objection, allowing them to refuse to perform terminations of pregnancies.^119^ This in some areas leads to a de facto impossibility for service users to have their pregnancy terminated. France became the first country to recently enshrine abortion as a constitutional right, though the practical consequences for healthcare professionals concerned remain to be seen.^120^ However, there seem to be other cases of challenging care situations which the studies included in our review did not mention as a course of moral distress, such as those pertaining to the application of informed consent and the assessment of decisional capacity. A recent review on midwives’ experiences in facilitating informed decision-making stated that a lack of informed decision-making leads to negative outcomes not only for service users but also for the midwives involved.^121^

Remarkably absent in the studies included in this review is the complete lack of attention to a specific cause of moral distress that has gained prominence in recent years, namely, ecological or environmental moral distress.^122–124^ Midwifery and obstetrics significantly contribute to the climate crisis due to the use of disposable materials and the utilization of nitrous oxide (N2O).^124^ Globally, N2O accounts for approximately 6% of global warming, with 1% of this attributed to its medical applications, including midwifery/obstetrics, dentistry, and pediatrics.^125^ Consequently, midwives face ethical dilemmata such as balancing the immediate benefits of certain procedures for service users against the long-term environmental impact, which could affect the well-being of both the birthing person and child.^126^ One possible explanation for the absence of the issues of ecological moral distress in the included studies may be that this phenomenon has only recently emerged as a topic of discussion and therefore was not sufficiently recognized by researchers and participants at the time the studies were conducted. At the same time, however, a number of midwifery associations have already published position papers on this subject, such as the International Confederation of Midwives and the German associations of midwives and of gynecologists.^127,128^ This highlights a significant research gap that warrants further investigation.

The definitional uncertainty and delineation of terms such as moral distress, moral injury, and moral discomfort are subjects of extensive debate, closely linked to challenges in measuring these phenomena.^129^ The valuable distinction proposed by Monteverde between situations, where individuals prevented from carrying out the right action (moral complicity) and situations in which moral action is burdensome due to conflicting moral values (moral complexity), draws attention to a crucial difference^3^: on the one hand, cases of moral complicity could be overcome—at least theoretically—through increasing resources, improving working conditions, enhanced education, critically scrutinizing power and knowledge structures within institutions, etc. Like these, they are rather results of political and societal choices regarding resource allocation. On the other hand, situations of moral complexity often present genuine dilemmata where there may never be an ideal solution. However, while the conceptual distinction between complexity and complicity holds significant value, it is important to recognize that they cannot always be neatly separated from each other. In reality, they are sometimes interconnected, blurring the lines between them in certain contexts. For example, the categorization of defensive practice in healthcare either as a result of moral complexity or of moral complicity presents significant challenges. Defensive practices seem to arise from a complex interplay of external pressures such as litigation fears, which may lead healthcare providers to practice defensively as a form of self-protection.^130^ The prevailing medical model, which often sidelines midwifery, might influence midwives to adopt more conservative practices. In certain contexts, moral complexity is evident as midwives navigate between adhering to their own professional ethics and the restrictive practices shaped by the healthcare system. This internalization of external factors blurs the lines between complicity and complexity, as practitioners may adopt these behaviors not purely from direct institutional mandates but also from a cultivated instinct to mitigate personal and professional risks. Thus, it becomes difficult to discern whether such practices are a response to the systemic flaws of the healthcare system or a personal strategy to navigate these flaws.

Last but not least, moral distress among midwives, particularly those early in their careers or those engaged in non-clinical roles such as research and academia, is often exacerbated by educational constraints and the varied landscape of professional opportunities across Europe. Although it has been over 25 years since the Bologna Agreement was implemented to harmonize higher education in Europe,^131^ the quality and scope of midwifery education still vary significantly from one country to another.^132^ For instance, in Italy, midwifery is recognized as a university degree, yet it offers limited opportunities for academic careers or leadership roles within the field of midwifery itself, as paradoxically governance of midwifery degree programs remains under the control of medical doctors.^133^ This can lead to a sense of professional limitation and moral distress for those who wish to advance within academic circles or have more autonomy in their practice. To note that in only 7 out of 29 European countries, a midwife holds a regulatory position within the Department of Health, indicating a significant role in shaping practices and policies directly affecting the profession. Yet, within these few countries a nursing director may assume this role, potentially affecting the autonomy and specific focus on midwifery in educational settings.^132,134^ These disparities underscore a crucial need for ongoing reform and alignment in midwifery education across Europe to ensure that all midwives have equal opportunities to advance their careers and reduce the moral distress associated with educational and professional constraints.

### Limitations

This scoping review has several limitations, the first of which pertains to the conceptual fuzziness surrounding moral distress. Due to the lack of universal consensus on the definition, we opted for a broad definition and included studies employing various definitions and terms, even those not explicitly using the term moral distress, to ensure inclusivity. Furthermore, we restricted our inclusion criteria to studies available in English, German, French, or Italian. Additionally, the overwhelming majority of studies originated from high-income countries, with a disproportionate representation from the European geographical area.^56^

## Conclusion

Despite projected national and international increases in demand of midwives,^33^ the midwifery workforce globally faces a crisis and is experiencing substantial strain, a trend expected to worsen in the coming years.^135^ Taken together moral distress not only burdens midwives but also impacts service users, children, and birth companions—and like that has adverse implications at a public health level. Our findings offer points of leverage to better monitor and alleviate moral distress among midwives, contributing to reducing attrition rates and ultimately improve care experiences for service users. Further research is essential to explore the issue of ecological distress and to develop and test evidence-based interventions aimed at alleviating moral distress among midwives and to evaluate the effects of both individual- and system-level interventions on midwives, intrapartum care, and service users’ outcomes.

## Supplemental Material

Supplemental Material - Causes of moral distress among midwives: A scoping review of international empirical literature Supplemental Material for Causes of moral distress among midwives: A scoping review of international empirical literature by Michael Rost, Caterina Montagnoli, and Johanna Eichinger in Nursing Ethics.

## Data Availability

The data and data collection sheets that support the findings of this study are available from the corresponding author upon reasonable request.*
