# Supplementary material for: Causes of moral distress among midwives: A scoping review
Source: Nurs Ethics. 2024 Sep 27;32(5):1382–405. doi: 10.1177/09697330241281498 (PMC12227815; doi:10.1177/09697330241281498)
Supplement: Supplemental Material - Causes of moral distress among midwives: A scoping review of international empirical literature [file sj-pdf-1-nej-10.1177_09697330241281498.pdf]

Appendix

Table 3. Underlying conflicts of identified causes of moral distress

Cluster	Sub-Cluster	Conflict[1]	Moral complicity vs. moral complexity	Study
(1) Societal disregard	**Health politics and law**	Importance of midwifery care vs. governmental deprioritization	Complicity	Callwood 2018
		Midwifery care vs. healthcare system changes at odds with quality midwifery care	Complicity	Thumm 2022
		Professional autonomy vs. oppressive state laws regulating practice	Complicity	Thumm 2022
	**Ignorance of authorities**	Right not to be subjected to violence vs. authorities' disregard and ignorance	Complicity	Boakye 2021
		Right not to be subjected to sexual harassment vs. lack of regulations on postings to other locations	Complicity	Jaffre 2021
		Truthfulness vs. administrations' failure to support midwives in face of negative online reviews	Complicity	Thumm 2022
	**Lack of recognition**	Importance of midwifery care vs. part of their work not socially acceptable	Complicity	Armour 2021
		Professional competence vs. trivialization of midwifery care	Complicity	Boakye 2021
		Professional competence vs. service users' dissatisfaction with midwifery care	Complicity	Thumm 2022
		Professional competence vs. lack of recognition by service users	Complicity	Harvie 2019
	**Violence**	Right not to be subjected to violence vs. violence and aggression	Complicity	Boakye 2021
(2) Contemporary birth culture	**Economization**	Midwifery care vs. imperatives of market-driven work environment	Complicity	Thumm 2022
		Appreciation of birth as an experience vs. birth distorted into a health-economic unit	Complicity	Thumm 2022
	**Socio-cultural norms**	Reproductive rights of service users vs. systematic disempowering of service users	Complicity	Narchi 2017
		Professional autonomy vs. physician-driven service users' perceptions and choices	Complicity	Surtees 2010
		Reproductive rights of service users vs. gender-related norms	Complicity	Boakye 2021
		Midwifery care vs. gender-related norms	Complicity	Boakye 2021
	**Medical model of care**	Professional autonomy vs. medicalized hospital setting	Complicity	Foster 2021
		Normal birth philosophy vs. medicalized maternity care	Complicity	Hadjigeorgiou 2013
		Avoidance of unnecessary and non-evidence based interventions vs. medicalization of birth	Complicity	Harvie 2019
		Normal birth vs. medical focus in birth	Complicity	Hood 2008
		Midwifery philosophy vs. medical philosophy	Complicity	Rice 2013
		Midwifery care vs. doctors' paranoia	Complicity	Surtees 2010
		Midwifery model vs. medical model	Complicity	Thumm 2022
		Professional autonomy vs. medicalized model of labour and birth	Complicity	Toohill 2018
		Midwifery led care vs. medically led services	Complicity	Foster 2021
	**Risk lens**	Midwifery care vs. risk-adverse environments	Complicity	Harvie 2019
		Natural birth vs. focus on the abnormal	Complicity	Hood 2008
		Midwifery care vs. climate of risk	Complicity	Surtees 2010
(3) Resources	**Staff**	Midwifery care vs. inadequately trained staff	Complicity	Mizuno 2011
		Midwifery care vs. inexperienced doctors	Complicity	Toohill 2019
		Midwifery care vs. poor midwifery skills	Complicity	Toohill 2019
	**Funding**	Midwifery care vs. budget cuts	Complicity	Harvie 2019
	**Time**	Paying attention to service users vs. lack of rest	Complicity	Hadjigeorgiou 2013
		Reciprocal supervision and exchange vs. lack of rest	Complicity	Hadjigeorgiou 2013

		Practicing values of midwifery care vs. high clinical activity levels	Complicity	Callwood 2018
		Reproductive rights of service users vs. workload pressures	Complicity	Foster 2021
		Midwifery care vs. heavy workload	Complicity	Hadjigeorgiou 2013
		Midwifery care vs. unrealistic workloads	Complicity	Harvie 2019
		Midwifery care vs. work overload	Complicity	Nunes 2016
		Midwifery care vs. intensified working conditions	Complicity	Reiger 2013
		Midwifery care vs. time pressures	Complicity	Callwood 2019
		Midwifery care vs. time pressures	Complicity	Groothuizen 2019
		Midwifery care vs. time pressures	Complicity	Hadjigeorgiou 2013
		Reproductive rights of service users vs. inadequate time	Complicity	Jaffre 2021
		Midwifery care vs. serious time pressures	Complicity	Oelhafen 2019
		Reproductive rights of service users vs. lack of time	Complicity	Oerlemans 2017
		Midwifery care vs. excessive workloads	Complicity	Boakye 2022
	**Infrastructure and materials**	Midwifery care vs. poor lighting system	Complicity	Addo 2020
		Midwifery care vs. limited resources	Complicity	Boakye 2021
		Midwifery care vs. lack of resources	Complicity	Boakye 2022
		Midwifery care vs. lack of equipment, space, resources	Complicity	Jaffre 2021
		Midwifery care vs. infrastructure problems	Complicity	Nunes 2016
		Midwifery care vs. limited resources	Complicity	Oelhafen 2019
		Midwifery care vs. lack of beds	Complicity	Reiger 2013
		Midwifery care vs. lack of resources	Complicity	Toohill 2019
	**Understaffing**	Midwifery care vs. lack of human resources	Complicity	Addo 2020
		Emergency care vs. lack of physicians	Complicity	Beck 2015
		Midwifery care vs. understaffing	Complicity	Toohill 2019
		Midwifery care vs. inadequate staffing	Complicity	Boakye 2021
		Midwifery care vs. reduced staffing levels	Complicity	Callwood 2018
		Midwifery care vs. staffing constraints	Complicity	Groothuizen 2019
		Midwifery care vs. shortage of staff	Complicity	Hadjigeorgiou 2013
		Midwifery care vs. chronic unsafe understaffing	Complicity	Harvie 2019
		Midwifery care vs. shortage of midwives	Complicity	Memmott 2022
		Reproductive rights of service users vs. shortage of midwives	Complicity	Memmott 2022
		Midwifery care vs. low staffing rate	Complicity	Mizuno 2011
		Midwifery care vs. insufficient quantity of providers	Complicity	Nunes 2016
		Midwifery care vs. inadequate staffing	Complicity	Oelhafen 2020
		Midwifery care vs. staff shortages	Complicity	Reiger 2013
		Midwifery care vs. low manpower	Complicity	Zolala 2019
**(4) Institutional characteristics**	**Facility culture**	Professional collaboration vs. "us versus them" culture	Complicity	Foster 2021
		Midwifery care vs. pressures to conform to facility culture	Complicity	Foster 2021
		Prioritizing service users vs. care as a "tick-box" exercise meeting institutional needs	Complicity	Harvie 2019
		Trust in birth vs. behavior imposed by institutional culture of fear	Complicity	Hood 2008
		Prioritizing service users vs. institution-focused environment	Complicity	Marsh 2020
		Reproductive rights of service users vs. technocratic and professional-centered system	Complicity	Narchi 2017
		Midwifery care vs. obstetricians' method of providing birthing care	Complicity	Nunes 2016

		Midwifery care vs. “eminence”-based care incl. questionable practices and values	Complicity	Oelhafen 2019
		Acknowledgment of midwifery care vs. no one cares about us	Complicity	Reiger 2013
		Individualized plan for care vs. hospital business plans, operational flows, provider scheduling	Complicity	Thumm 2022
		Birth as a transformative experience vs. culture of dehumanization of birth	Complicity	Thumm 2022
		Culture of constructive critique vs. culture unsupportive of raising concerns	Complicity	Callwood 2018
		Midwifery care vs. inevitably compromised care	Complicity	Callwood 2018
		Midwifery care vs. hospital system resistant to change	Complicity	Harvie 2019
		Naming unacceptable behavior vs. culture of ignorance	Complicity	Marsh 2020
	**Guidelines, policies, and protocols**	Prioritizing service users vs. loyalty to guidelines, evidence, interprofessional relationships	Complicity	Fontein-Kuipers 2018
		Professional autonomy vs. organizational policy	Complicity	Foster 2021
		Prioritizing service users vs. hospital guidelines and protocols	Complicity	Hadjigeorgiou 2013
		Prioritizing service users vs. policies, protocols, guidelines	Complicity	Harvie 2019
		Professional autonomy vs. guidelines and protocols	Complicity	Hood 2008
		Prioritizing service users vs. guidelines and increase in interventions	Complicity	Hood 2008
		Prioritizing service users vs. policies and guidelines	Complicity	Jefford 2022
		Professional autonomy vs. policies	Complicity	Reiger 2013
		Professional autonomy vs. institutional policies	Complicity	Zolala 2019
		Professional competence vs. policies requiring obstetricians signing medical records	Complicity	Zolala 2019
		Accountability of midwife entering perinatal data vs. entering data for others	Complicity	Craswell 2014
		Maximizing time spent with service users vs. time spent with electronic medical records	Complicity	Thumm 2022
**(5) Interprofessional relationships**	**Unsupportive climate**	Midwifery care vs. negative role models and mentors	Complicity	Callwood 2019
		Midwifery care vs. unsupportive physician supervision	Complicity	Thumm 2022
		Midwifery care vs. unsupportive attitudes by colleagues	Complicity	Toohill 2019
		Midwifery care vs. disorganization	Complicity	Jaffre 2021
		Midwifery care vs. toxic culture	Complicity	Boakye 2021
		Midwifery care vs. arguing with medical staff	Complicity	Hood 2008
	**Disrespect and trivialization**	Midwifery care vs. not valued, respected, appreciated by colleagues	Complicity	Harvie 2019
		Midwifery care vs. undervalued	Complicity	Hood 2008
		Midwifery care vs. lack of recognition and prejudices	Complicity	Jaffre 2021
		Midwifery care vs. hostile attitudes by colleagues	Complicity	Toohill 2019
		Midwifery care vs. disrespect from colleagues	Complicity	Addo 2020
		Midwifery care vs. ridicule and rudeness by other team members	Complicity	Boakye 2021
		Midwifery care vs. trivialization	Complicity	Boakye 2021
		Midwifery care vs. negative treatment and devalued	Complicity	Foster 2021
		Midwifery care vs. disrespect	Complicity	Hadjigeorgiou 2013
		Midwifery care vs. disrespect	Complicity	Toohill 2019
	**Oppressive power asymmetries**	Professional autonomy vs. physicians in charge	Complicity	Beck 2022
		Professional autonomy vs. interprofessional power struggles and oppressive environments	Complicity	Boakye 2021
		Midwifery care vs. hierarchy	Complicity	Callwood 2018
		Rights-based care vs. hierarchical culture	Complicity	Foster 2021
		Midwifery care vs. hierarchical and restrictive culture	Complicity	Foster 2021
		Midwifery care vs. pressures, hierarchy, authoritarianism	Complicity	Hadjigeorgiou 2013
		Professional autonomy vs. hierarchy and power struggles	Complicity	Harvie 2019

		Right to be free from sexual harassment vs. sexual solicitation by superiors	Complicity	Jaffre 2021
		Midwifery care vs. hierarchical and patriarchal environment	Complicity	Jefford 2022
		Midwifery care vs. hegemonic attitude by physicians	Complicity	Nunes 2016
		Midwifery care vs. hierarchical system	Complicity	Oelhafen 2019
		Midwifery care vs. asymmetries of power and authority	Complicity	Oelhafen 2020
		Midwifery care vs. hierarchical authoritative environment	Complicity	Thumm 2022
		Midwifery care vs. bullying	Complicity	Toohill 2019
	**Epistemic disavowal**	Professional competence vs. ignorance	Complicity	Addo 2020
		Professional competence vs. lack of epistemic authority and disqualification	Complicity	Boakye 2021
		Professional competence vs. perceived as less valid and questioned	Complicity	Foster 2021
		Professional competence vs. ignorance	Complicity	Hadjigeorgiou 2013
		Professional competence vs. silenced	Complicity	Harvie 2019
		Professional competence vs. having no voice	Complicity	Jefford 2022
		Professional competence vs. opinions not accepted	Complicity	Nunes 2016
		Professional competence vs. lack of trust in competence	Complicity	Oelhafen 2020
**(6) Interpersonal mistreatment in birth**	**Physical abuse**	Rights-based care vs. necessity of violence	Complexity	Jaffre 2015
		Rights-based care vs. tolerance of abuse by other providers	Complicity	Narchi 2017
		Rights-based care vs. being complicit in violent care	Complicity	Toohill 2019
	**Verbal abuse**	Rights-based care vs. harsh language, insults	Complicity	Jaffre 2021
		Rights-based care vs. harsh language	Complicity	Rice 2013
		Rights-based care vs. inappropriate language, pressure, lies, rudeness	Complicity	Toohill 2019
		Rights-based care vs. lies	Complicity	Beck 2022
		Rights-based care vs. rough approaches	Complicity	Beck 2015
	**Poor rapport**	Rights-based care vs. lack of respect	Complicity	Jaffre 2021
		Rights-based care vs. rudeness	Complicity	Reiger 2013
		Rights-based care vs. providers' lack of respect	Complicity	Callwood 2019
		Rights-based care vs. limited autonomy	Complicity	Oelhafen 2019
		Rights-based care vs. not respecting service user's demand to stop vaginal examination	Complicity	Toohill 2019
		Rights-based care vs. unwanted epidural	Complicity	Rice 2013
	**Failure to meet professional standards of care**	Rights-based care vs. limited choice	Complicity	Beck 2022
		Rights-based care vs. inadequate consent	Complicity	Foster 2021
		Rights-based care vs. lack of choice	Complicity	Hadjigeorgiou 2013
		Rights-based care vs. little or disrespect of informed choice	Complicity	Toohill 2019
		Rights-based care vs. lack of privacy	Complicity	Toohill 2019
		Rights-based care vs. deception	Complicity	Zolala 2019
		Rights-based care vs. unnecessary cesarean sections and inductions	Complicity	Beck 2022
		Rights-based care vs. unnecessary interventions	Complicity	Foster 2021
		Rights-based care vs. unnecessary cesarean sections	Complicity	Nunes 2016
		Rights-based care vs. unnecessary episiotomy and cascade of medical interventions	Complicity	Rice 2013
		Rights-based care vs. unnecessary interventions	Complicity	Thumm 2022
		Rights-based care vs. unnecessary surgical delivery, inductions, and rupture of membranes	Complicity	Toohill 2019
		Rights-based care vs. futile care	Complicity	Zolala 2019
		Rights-based care vs. unnecessary interventions	Complicity	Hood 2008

(7) Defensive practice	Fear of litigation	Midwifery care vs. fear of litigation	Complexity	Fontein-Kuipers 2018
		Midwifery care vs. fear of litigation	Complexity	Groothuizen 2019
		Midwifery care vs. fear of lawsuits	Complexity	Guidera 2021
		Advocating for woman vs. fear of litigation	Complexity	Hood 2008
		Midwifery care vs. fear of litigation	Complexity	Robertson 2016
		Midwifery care vs. fear of litigation	Complexity	Surtees 2010
		Midwifery care vs. fear of being sued	Complexity	Toohill 2019
		Trusting interprofessional relationships vs. fear of being sued	Complexity	Toohill 2019
	Further emotional reasons	Midwifery care vs. distrust in service users	Complexity	Surtees 2010
		Midwifery care vs. anxiety and depression	Complexity	Kerkman 2019
		Midwifery care vs. heightened index of suspicion	Complexity	Beck 2015
(8) Challenging care situations	Midwifery care	Autonomy of service users to terminate pregnancy vs. right to life of fetus	Complexity	Cignacco 2002
		Autonomy of service users to terminate pregnancy vs. right to life of fetus	Complexity	Foster 2021
		Autonomy of service users to terminate pregnancy vs. danger of eugenic values and slippery slope	Complexity	Garel 2002
		Autonomy of service users to terminate pregnancy vs. right to life of fetus	Complexity	Garel 2007
		Autonomy of service users to terminate pregnancy vs. right to life of fetus	Complexity	Lindström 2011
		Autonomy of service users to terminate pregnancy vs. right to life of fetus	Complexity	Mizuno 2011
		Autonomy of service users to terminate pregnancy vs. right to life of fetus	Complexity	Mizuno 2013
		Autonomy of service users to terminate pregnancy vs. right to life of fetus	Complexity	Oelhafen 2020
		Autonomy of service users to terminate pregnancy vs. right to life of fetus	Complexity	Zareba 2020
		Autonomy of service users to terminate pregnancy vs. right to life of fetus	Complexity	Zolala 2019
		Autonomy of service users to have elective interventions vs. avoidance of unnecessary interventions	Complexity	Oelhafen 2020
		Autonomy of service users to hold unrealistic expectations vs. midwifery care	Complexity	Thumm 2022
		Inform about newborn screening and diseases vs. reassuring service users	Complexity	Oerlemans 2017
		Autonomy of service users to make decisions not recommended vs. midwifery care	Complexity	Toohill 2019
		Autonomy of service users to have elective cesarean sections vs. avoidance of unnecessary interventions	Complexity	Zolala 2019
	Circumstances of service users	Assuming extra-job responsibilities for impoverished service users vs. managing own finances	Complexity	Boakye 2021
		Midwifery care vs. impoverished service users not being able to access care	Complicity	Boakye 2021
		Midwifery care vs. impoverished service users not being able to pay	Complicity	Zolala 2019
	Pandemic	Midwifery care vs. pandemic restrictions	Complicity	Fumagalli 2022
		Midwifery care vs. infection control protocols	Complicity	Kane 2022
		Job security and finances vs. avoidance of infection	Complexity	Kane 2022
		Midwifery care vs. fear of transmission to service users	Complexity	Memmott 2022
		Midwifery care vs. pandemic restrictions	Complicity	Memmott 2022
	Child protection	Removal of baby at birth in the best interest of baby vs. centering service users in midwifery care	Complexity	Marsh 2020

Note. [1]For conflicts between midwives' moral values and external constraints that compromise their moral agency (complicity), the first part refers to the moral value of the midwife and the second part to the external constraint; the remaining conflicts refer to conflicts between two or more moral values of the midwife (complexity). Moreover, for conflicts between midwives' moral values and external constraints, external constraints are mainly presented (i.e., terminology) as reported in the respective article.
